# Editorial: Perivascular Adipose Tissue (PVAT) in Health and Disease

**DOI:** 10.3389/fphys.2018.01004

**Published:** 2018-07-30

**Authors:** Stephanie W. Watts, Maik Gollasch

**Affiliations:** ^1^Pharmacology and Toxicology, Michigan State University, East Lansing, MI, United States; ^2^Experiment and Clinical Research Center, Medical Clinic of Nephrology and Internal Intensive Care, Charité Universitätsmedizin Berlin, Berlin, Germany

**Keywords:** perivascular adipose tissue, cardiovacsular Disease(s), vascular dysfunction, immune system, adipose tissue

The field of perivascular adipose tissue (PVAT) research began with a simple and elegant study published in 1991, and the year 2018 finds us with substantially greater knowledge that this adipose tissue around a blood vessel possesses profound abilities to modify vascular tone. Soltis and Cassis (1991) performed the original studies on the isolated rat thoracic aorta, demonstrating that removal of the PVAT around the aorta shifted the contraction to NE to the left in a way that was functionally dependent on NE uptake. These studies were the first to suggest the PVAT was more than structural support, and were followed by an important study in 2002 demonstrating that PVAT from healthy subjects contains factors that directly relaxed contracted arteries (Lohn et al., 2002). Since then these and other findings resulted in the phrase of PVAT being “anti-contractile” in health. PubMed lists over 700 publications on perivascular adipose tissue (search June 17, 2018). The contributions made in this Frontiers Research Topic are significant because they add to each of the considerations that have been given to PVAT relative to its role in vascular function.

The cell types that constitute PVAT appear to be different in different sites of the body, with the adipocyte type in PVAT including brown, beige and white adipocytes (Pfeifer et al.). This was elegantly discussed by Pond with a historical perspective on accuracy and artistry in Western Europe (Pond). Adipocyte type appears not be static with the finding that the browning of PVAT is associated with aging (Kong et al.). The adipocyte is but one cell type that could contribute to PVAT being supportive of vessel health, or contributing to vascular dysfunction in disease.

The ability of PVAT to be beneficial to a vessel in health is further supported by the observations that PVAT produces other relaxant substances such as palmitic acid methyl ester (Wang et al.), harbors atheroprotective B cells (Srikakulapu et al.), and provides mitochondrial function that blunts atherosclerosis (Xiong et al.). In the human, preservation of PVAT improves saphenous vein graft function with the “no-touch” technique (Vestergaard et al.), a finding that encourages keeping the PVAT layer intact in isolated veins before grafting. PVAT works in concert with the vessel it surrounds; this relationship is biologically important given the importance of the molecule insulin receptor substrate 2 (IRS2) to modify insulin-induced changes in vasomotor tone in a PVAT dependent manner (Turaihi et al.). The mechanisms by which PVAT achieve an anti-contractile effect (e.g., mediators, signal transduction pathways) may not be the same in the human and rodent. Thus, the fields continued push to study human vessels remains important.

A significant reason for investigating PVAT is the loss of the anticontractile effect of PVAT in cardiovascular diseases, as reviewed by da Costa et al and demonstrated specifically in the condition of a high fat diet (Almabrouk et al.), in atherosclerosis (Tanaka and Sata), in metabolic syndrome (da Costa et al.), and other non-atherosclerotic vascular diseases (Horimatsu et al.). By contrast to these findings that suggests PVAT may play a role in the pathology of cardiovascular diseases, work by Baltieri suggests that PVAT may be protective of endothelial function and insulin-induced vasodilation of the hypercholesterolemic mouse (Baltieri et al.). There remains the question of when, temporally, changes in PVAT occur relative to the disease proper, such that interventions at the level of PVAT could influence disease outcome.

This Frontiers Research Topic in PVAT is a view into the rich places of research that have yet to be tapped relative to understanding the role PVAT plays both in health and disease. We are grateful to our contributors for sharing their important work.

## Author contributions

SW drafted the editorial, and MG read and modified editorial.

### Conflict of interest statement

The authors declare that the research was conducted in the absence of any commercial or financial relationships that could be construed as a potential conflict of interest.

## References

[B1] Lohn M. Dubrovska G. Lauterbach B. Luft F. C. Gollasch M. Sharma A. M. (2002). Periadvential fat releases a vascular relaxing factor. FASEB J. 16, 1057–1063. 10.1096/fj.02-0024com 12087067

[B2] Soltis E. E. Cassis L. A. (1991). Influence of perivascular adipose tissue on rat aortic smooth muscle responsiveness. Clin. Exp. Hypertension A 13, 277–296. 2065467 10.3109/10641969109042063

